# Every Detail Matters. That Is, How the Interaction between Gα Proteins and Membrane Affects Their Function

**DOI:** 10.3390/membranes11030222

**Published:** 2021-03-20

**Authors:** Agnieszka Polit, Paweł Mystek, Ewa Błasiak

**Affiliations:** Department of Physical Biochemistry, Faculty of Biochemistry Biophysics and Biotechnology, Jagiellonian University, Gronostajowa 7, 30-387 Kraków, Poland; pawel.mystek@uj.edu.pl (P.M.); ewa.blasiak@uj.edu.pl (E.B.)

**Keywords:** G proteins, membrane domains, signal transduction, G-protein-coupled receptors, GTP-binding proteins, lipids

## Abstract

In highly organized multicellular organisms such as humans, the functions of an individual cell are dependent on signal transduction through G protein-coupled receptors (GPCRs) and subsequently heterotrimeric G proteins. As most of the elements belonging to the signal transduction system are bound to lipid membranes, researchers are showing increasing interest in studying the accompanying protein–lipid interactions, which have been demonstrated to not only provide the environment but also regulate proper and efficient signal transduction. The mode of interaction between the cell membrane and G proteins is well known. Despite this, the recognition mechanisms at the molecular level and how the individual G protein-membrane attachment signals are interrelated in the process of the complex control of membrane targeting of G proteins remain unelucidated. This review focuses on the mechanisms by which mammalian Gα subunits of G proteins interact with lipids and the factors responsible for the specificity of membrane association. We summarize recent data on how these signaling proteins are precisely targeted to a specific site in the membrane region by introducing well-defined modifications as well as through the presence of polybasic regions within these proteins and interactions with other components of the heterocomplex.

## 1. Introduction

The correct development of cells depends on how they respond to various environmental factors and how they develop stress tolerance. Cells possess several signal transduction mechanisms. The basic one involves receptors that perceive the signal and trigger a response in the form of secondary messengers. Various mechanisms are responsible for signal conversion from the primary to the secondary messengers, among which G proteins play a significant role (review: [1,2]). G proteins belong to numerous and diverse family of proteins binding guanine nucleotides and possess guanosine triphosphate (GTP) hydrolase activity. As the name suggests, G protein heterotrimer consists of three different G protein subunits: Gα, Gβ, and Gγ. The first of these subunits determines the name and, more importantly, properties of the heterotrimer (review: [3,4]). A group of small monomeric proteins that bind to and hydrolyze GTP, such as Rho and Ras proteins, is also included in the G protein family (review: [5]), but this article will not focus on such proteins.

Traditionally, the designation of heterotrimeric G proteins is derived from the name of the α subunit of G proteins. In humans, Gα subunits are encoded by 16 genes [6]. Some of the subunits encoded by these genes undergo alternative splicing, and therefore, over 20 isoforms of the Gα subunits have been shown to exist to date. Each of them consists of two domains. The first one, a highly conserved GTPase domain, is responsible for binding and hydrolysis of GTP. The second one, an alpha helical domain, is varied and, together with C-terminal end, responsible for G protein-coupled receptor (GPCR)/effector specificity determination [7,8,9]. Guanine nucleotide is bound in the space between these domains [10,11]. In its inactive form, the Gα protein binds guanosine diphosphate (GDP), and in its active form, it binds GTP. Comparison of structures of Gα bound with GDP and GTP showed the presence of elastic regions, so-called switches, that become ordered in active conformation in which the protein is bound with GTP [10]. GTPase domain can bind not only guanosine nucleotides but also to Gβγ complexes, GPCRs, and effector proteins. Although there is incomplete knowledge on the structures of N- and C-termini of the Gα subunit, they probably play a role in the activation and determination of specificity toward other proteins as well as association with the membrane. According to Gα sequence homology and function, four families are distinguished: G proteins responsible for the activation of adenylate cyclase (Gαs), proteins inhibiting adenylate cyclase (Gαi/o), proteins responsible for the activation of phospholipase C (Gαq/11), and proteins responsible for the stimulation of Na^+^/H^+^ antiport, phospholipase D, and protein kinase C–group Gα12/13 [12]. Further division within the families of Gα proteins is presented in Table 1.

There are five different Gβ (Gβ_1-5_) and twelve different Gγ (Gγ_1-5_ and Gγ_7-13_) proteins in humans encoded by different genes [6,13]. They are closely associated, and the Gβγ dimers are probably formed co-translationally; thus, they are often regarded as one unit [14,15]. While Gβ_1-4_ are found in all human cells, the Gβ_5_ subunit is found mainly in the human brain. The Gγ subunit sequences are the most heterogeneous of the three subunit families. The C-terminus of this subunit can undergo prenylation, which increases its affinity to the plasma membrane. -N terminal helixes 1 and 2 of the Gγ protein were indicated as being responsible for interacting with Gβ first [11]. However, Wall et al.’s study suggests that additional interactions occur, also involving amino acids present in the C-terminal region of the Gγ subunit [16]. The nascent, very stable Gβγ complex binds to the hydrophobic pocket of the inactive Gα-GDP protein. The βγ complex itself can also influence downstream effectors such as adenylyl cyclases, phospholipase Cβ, and K^+^ and voltage-gated channels [17] (review: [18]). Several studies have reported that a particular receptor requires the participation of a specific Gβγ complex. Although in vitro studies have shown that most Gα subunits can associate with most Gβγ dimers, there is no comprehensive knowledge on the combination of Gα, Gβ, and Gγ in heterocomplexes that exist in vivo [14].

Unlike protein tyrosine and serine/threonine kinase receptors, which have intrinsic catalytic activity, GPCRs do not have enzymatic activity per se but are linked to Gα proteins, which mediate signal transduction. This dynamic signal transmission from the cell’s environment to the effector molecules existing inside the cell is a consequence of cyclical activation and inactivation of heterotrimeric G proteins. As mentioned above, in its inactive state, the Gα subunit is bound to GDP and is associated with the residual Gβγ subunit. Activation of the GPCR by a specific ligand induces conformational changes within the receptor’s intracellular loops. These changes enable interaction and activation of the Gαβγ heterotrimer. The complex, consisting of three elements—ligand, receptor, and the G protein—undergoes consecutive conformational changes in the GTPase region of the Gα subunit. These changes cause the replacement of GDP by GTP in the heretofore inactive Gα subunit. The activated G protein dissociates from the receptor, and its subunits disconnect because of conformational changes within the Gα hydrophobic pocket [19]. The Gα-GTP protein and the Gβγ complex can now regulate the activity of certain intracellular effector proteins, causing changes in the level of secondary transmitters and regulating strict signaling pathways. Intrinsic hydrolase activity of the Gα subunit converts GTP to GDP, and this step allows the cycle to end with the reconstruction of the Gαβγ complex. Classically, it is believed that the processes described above occur in the plasma membrane and are terminated by receptor desensitization and internalization mediated by β-arrestins. It is now known that G-protein activation is not restricted to the plasma membrane and that receptors can be active after endocytosis. Some GPCRs can retain or recover the ability to activate G proteins after internalization and subsequently activate not only G proteins associated with the cell membrane, but also those located in the cytoplasm, within endosomes or membrane compartments in the biosynthetic pathway (review: [20,21]). This fact makes signaling through G proteins even more complicated due to differences in protein-partner availability and differences in the environment in which transduction elements are located.

Signaling from GPCRs through heterotrimeric G proteins requires proper localization of these proteins on the cytoplasmic side of the membrane. This can be accomplished primarily through lipid modifications. Lipidation of proteins can be of four types: glypiation, cholesterylation, prenylation, and fatty acylation. Of these lipid modifications, prenylation (geranylgeranylation and farnesylation) and acylation (S- and N-palmitoylation, N-myristoylation) of protein elements of the GPCRs signaling pathway play vital roles in their function and regulation [22]. The function of lipidation is not limited to docking proteins in the membrane, but it also restricts the diffusional mobility of proteins from three to two dimensions [23] and enables protein–lipid and protein–protein interactions [24,25]. GPCRs signaling is organized in discrete membrane compartments and dynamically connected through membrane trafficking of signaling molecules [20,26,27,28]. As signal transduction is a highly efficient and spatially restricted sequence of events, G proteins fulfill their role by having multiple membrane-targeting motifs. Apart from lipidation, other factors responsible for membrane targeting of these proteins are interactions of polybasic clusters with lipids as well as protein–protein interactions between subunits that are part of the trimer and with other signaling proteins.

This review aims to provide an overview of the mechanisms of Gα subunits’ interaction with lipids and factors responsible for the specificity of membrane association. This knowledge is very important as it shows how different signaling proteins can be precisely targeted at a predestined location by incorporating well-defined modifications. We focused on the Gα subunits as new data revealed considerable diversity in membrane trafficking properties of closely related Gα subunits [29,30]. Furthermore, it has been found that the membrane distribution of heterotrimeric G proteins is not dependent only on Gβγ, as was commonly believed. Gα itself also acts as a crucial modulator of the membrane localization of the full complex [31].

## 2. What Makes Gα Dock to the Membrane?

When considering how Gα subunits bind to the plasma membrane and what affects their membrane localization, it is important to understand that multiple membrane-binding mechanisms function together in this case. One of the reasons why the Gα binds to the membrane is a lipid anchor at the N-terminus of the Gα subunit and the second is ionic interaction driven by the charge–charge attraction between the cluster of basic residues within Gα and acidic phospholipids. Thus, two (or more, if considering heterotrimeric complex) binding signals synergize to provide a high affinity and specificity of Gα interaction with membrane lipids.

The two-signal hypothesis, originally introduced to explain the membrane attachment of Ras proteins, explains the role of lipidation in membrane anchorage of heterotrimeric G proteins [32]. In this model, the single lipid modification is insufficient for stable membrane attachment. The desired effect may be the result of a combination of lipid anchors, lipid-binding motifs, or charged regions. All Gα subunits are lipidated in the region of the N-terminal helix, which is oriented parallel to the cell membrane and is responsible for proper localization and interactions with the Gβγ dimer. These functions are achieved by lipid anchors and/or amino acid residues on the opposite sides of the helix. Molecular and atomistic details of such an interaction are missing. Most available crystal structures of monomeric Gα do not contain this part of the molecule, because often the Gα-GDP used for crystallization lacks an N-terminus [33,34,35,36]. By using a secondary structure prediction algorithm (PSIPRED algorithm), N-terminus of Gα subunits adopts an α helix structure with a positively charged surface facing inward to the surface of cellular membranes [37,38,39]. However, in the Gαi_1_ crystal structure, residues 1 to 8 remain disordered. The next eight residues fold into a three-turn αhelix terminated with a single, four-residue 3_10_ helix [40]. In the crystal structures of the heterotrimeric complexes, the lipidated N-terminus of Gα has an extended helical conformation that lies along the surface of the polar head groups of the lipids and is stabilized by interactions with Gβγ [10,41].

In principle, the Gα and Gγ subunits, but not the Gβ subunit, undergo a range of lipid modifications. All the Gα proteins are either palmitoylated and/or myristoylated. While at least one palmitoyl lipid anchor is present on all Gα subunits, except for Gαt and Gαg, the presence of the myristoyl group differs across all Gα families and even among one family.

### 2.1. Palmitoylation

Apart from Gαt and Gαg subunits, all Gα subunits undergo palmitoylation. This modification is an attachment of saturated acyl lipid of 16-carbon palmitic acid (16:0) to the cysteine residue in the amino acid sequence by thioester bond (S-palmitoylation) [42,43]. A consensus sequence for this protein modification has been loosely described. As Salaun and colleagues report in their paper, cysteines that undergo this type of modification show some common features. First, they are usually located close to myristoylation and prenylation sites. The second feature uniting palmitoylated cysteines is their surroundings, which are usually basic or hydrophobic amino acids. The third is the occurrence of modified cysteines within transmembrane domains or cytoplasmic regions flanking them [44]. There is also evidence that palmitoylation is determined by secondary structure rather than primary amino acid sequence [45]. Protein palmitoylation frequently requires prior membrane attachment as the enzymes that catalyze this reaction are integral membrane proteins [46].

Interestingly, many proteins of the GPCR signaling pathway undergo this modification. Palmitoyl moiety can be attached to certain GPCRs [47], Gα subunits [48,49], regulator of G protein signal (RGS) and its binding protein–R7BP [25,50], phosphodiesterase [51], and G protein-coupled receptor kinases (GRKs) [52,53,54]. It seems that the importance of palmitoylation is not limited to membrane localization. It is possible that palmitoylation regulates the desired colocalization of interacting proteins. In this context, the reversibility of this modification seems to be particularly interesting. Cyclic palmitoylation-depalmitoylation of individual system components can act as a regulatory mechanism, like phosphorylation, by colocalizing or separating the system components. (review: [55]). However, such regulation is not straightforward. Among the palmitoylated receptors, we can distinguish those where the modification has a significant effect on the interaction with cognate G proteins, such as β_2_-adrenergic or M2 muscarinic acetylcholine receptor. On the other hand, there are those receptors for which no relationship between lipidation and G protein coupling has been found, such as dopamine D_1_ or adenosine A1 receptor (review: [56]). Many studies on palmitoylated proteins and peptides have indicated that such modification targets the signal to lipid raft-like domains [57,58,59]. In the nervous system, protein palmitoylation can make a crucial contribution to brain development, synaptic transmission, and trafficking within the neuron terminals (reviews: [42,60]. Since palmitoylation regulates the functions of proteins that control neuronal differentiation, axonal pathfinding, filopodia formation, and trafficking, these processes require this modification.

**Figure 1:**
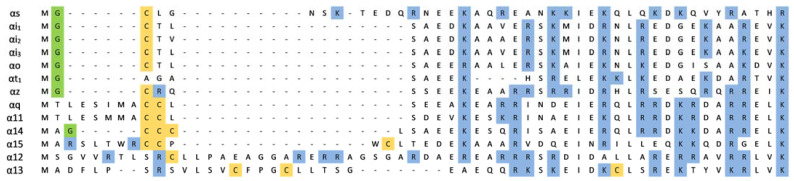
Sequence alignment of the N-terminal region of selected human Gα subunits. The residues are colored to indicate their modifications or properties: green—N-myristoylated glycine residue (for Gαs N-palmitoylated glycine); orange—S-palmitoylated cysteine; blue—residues with a positive charge.

Subunits of Gαq/11 and Gα12/13 families and Gαolf subunit undergo solely palmitoylation. The Gαolf and Gα12 subunits have one modified cysteine residue in their sequence; other subunits of mentioned families may be modified at two or even three amino acids (Figure 1). The single anchor of the Gα12 subunit is required for membrane binding and proper coupling to the thrombin receptor [61]. However, its cytoplasmically localized non-palmitoylated mutant can reconstitute a membrane-surface-localized heterotrimer when coexpressed with Gβγ dimer [61]. For Gα13, which is palmitoylated thrice, two palmitoylation sites were examined and shown to be necessary for attachment and receptor-G protein interaction [62]. Two palmitoyl anchors are significant for the localization of Gαq and signal transmission followed by receptor activation [49]. Yet, a single anchored Gαq mutant is capable of efficient signal transduction from the α2-adrenoreceptor [49]. During the palmitoylation–depalmitoylation cycle, the protein commutes between the plasma membrane and the Golgi [63].

In addition to S-palmitoylation, the rest of palmitoyl acid can be attached to the N-terminal glycine. This reaction occurs by amide bonding [64] and is permanent. The Gαs subunit is unique among the other Gα subunits. This subunit has two lipid anchors, similar to the members of the Gαi subfamily, both of which are palmitoyl (N- and S-palmitoyl anchor). Initial reports on the role of palmitoylation of the Gαs subunit provide contrary information. Wedegaertner and colleagues determined that non-S-palmitoylatable Gαs mutant localizes mainly in the cytosol with negligible cAMP generation after receptor stimulation [49]. On the other hand, other researchers demonstrated that the lack of S-palmitoylation and the deletion of the following helix region do not disrupt plasma membrane localization of Gαs [65,66]. The cell localization of Gαs changes in response to receptor stimulation. This subunit undergoes rapid depalmitoylaton potentiated by receptor activation, inducing detachment of the Gαs subunit from the plasma membrane [67].

### 2.2. N-Myristoylation

The Gαi subfamily members, namely Gαi_1-3_, Gαo_1-2_, and Gαz, besides undergoing S-palmitoylation, are N-myristoylated [68]. Lipidation of the Gαg subunit is not well described, but based on the sequence similarity of the Gαt and Gαg proteins, it can be hypothesized that both proteins are solely myristoylated [69]. Myristoylation is a co-translational modification that adds myristic acid (C14) to the N-terminal glycine residue through peptide bonding [70]. This process is considered to be irreversible, and it requires the removal of initiation methionine and the presence of serine or threonine in the sixth position in the primary sequence [71].

Gαi mutations preventing N-myristoylation resulted in a cytoplasmic distribution of this protein. However, membrane localization of mutants was restored after Gβγ dimer co-expression [72,73]. A similar effect was observed for the mutant with impaired palmitoylation; again, Gβγ co-expression restored the membrane localization of the complex [74]. These results indicate that the myristoylation of the Gαi subunit is not required for palmitoylation and that the membrane localization signal provided by the Gβγ dimer might be sufficient for palmitoylation. For the Gαz subunit, prior myristoylation might be required for palmitoylation because the non-myristoylated mutant is soluble and fails to incorporate palmitate. Coexpression with Gβγ only partially restores membrane attachment and palmitoylation of Gαz [75].

The myristoylation process also affects the interaction of Gα subunits with effector proteins. The non-myristoylated mutant of the Gαz subunit is unable to activate mitogen-activated protein kinase (MAPK) [76]. Similar dysfunctions were observed for Gαi subunits. Mutation of the myristoylation site in the sequence of Gαi_2_ results in the inability to activate p42-mitogen-activated protein kinase and to inhibit the activity of adenylyl cyclase [77]. For Gαi_1_, an identical mutation also results in the failure of inhibition of adenylyl cyclase 5 [78]. Interestingly, an in vitro study on the Gαi_1_ subunit showed prominent differences between conformations of myristoylated and non-myristoylated forms, thus suggesting a critical role of lipidation in the structure and function of these subunits [79]. Recent molecular dynamics simulations demonstrated the stability of the myristoyl moiety due to a unique hydrophobic pocket within the Ras domain and the N-terminal region of the Gαi_1_ subunit and also showed the influence of myristoylation on the conformation of the subunit [80]. The observed conformational differences, resulting from myristoylation, explain the opposite effect of the Gαs and Gαi subunits on adenylyl cyclase. This aspect has been problematic because Gαs and non-myristoylated Gαi are structurally very similar [81].

### 2.3. Prenylation

Although we exclusively focused on Gα subunits, Gγ subunit lipidation should also be mentioned, as it is important for efficient receptor signal transduction. Prenylation is a multistage modification that involves covalent attachment of the hydrophobic farnesyl (C15) or geranylogeranyl (C20) moiety by the thioether bond. Prenylation is an irreversible modification of cysteine residue within the CAAX motif, which determines the specificity of the modification. Prenylation is followed by cleavage of the last three amino acid residues, and prenylcysteine is then methylated [82]. Farnesylation occurs in Gγ_1_, Gγ_9_, and Gγ_11_ subunits; the remaining Gγ subunits undergo geranylogeranylation [83].

It was found that prenylation alone might be insufficient for stable membrane docking, but along with S-acylation of Gα subunit, it can stabilize the heterotrimer–plasma membrane interaction [84,85]. Therefore, complexing Gβγ constitutes a second membrane docking signal for singly modified Gα subunits [21]. Prenylation of the Gγ subunit is required not only for proper Gβγ membrane localization but also for effective heterotrimer Gαβγ formation and for particular interaction with effector proteins such as adenylyl cyclase or phospholipase Cβ2 [86,87]. Farnesyl anchors drive membrane proteins to non-raft domains [88,89]. Although prenylation modification is permanent, the methylation of the C-terminal prenylcysteine is reversible and might be regulated. Methylation, by neutralizing the negative carboxylate group at the C-terminus of the Gγ subunits, might affect interaction with effector proteins or charged lipid head groups on the plasma membrane.

### 2.4. Polybasic Motifs

In addition to lipid modifications of the Gα subunits, a polybasic motif at the N-terminal helical region has been shown to influence their membrane binding and localization as well as interaction with the Gβγ dimer [30,37,90,91]. Polybasic motifs are present in a multitude of plasma membrane-targeted proteins, including domains specialized in interacting with cell membranes [92,93,94,95]. A positively charged amino acids cluster can be located in the primary sequence but might also result from a three-dimensional clustering of more distant amino acids. These regions contain a combination of lysine and arginine that cooperate with aromatic/hydrophobic residues and/or lipid modifications. The presence of hydrophobic moieties is critical for enhancing the binding of the polybasic domain to the lipid bilayer [93]. As has been shown for small GTPases, most of the polybasic clusters contain two or three subclusters, each spanning approximately four to five amino acids [92,93]. Removing one of the flanking subclusters abolished plasma membrane targeting, leading to the proposal that association results from the additive binding of individual subclusters [93]. These positively charged protein regions interact electrostatically with the negatively charged phospholipid head groups on the inner leaflet of the plasma membrane and thus favor specific targeting of proteins to the plasma membrane [96].

**Figure 2:**
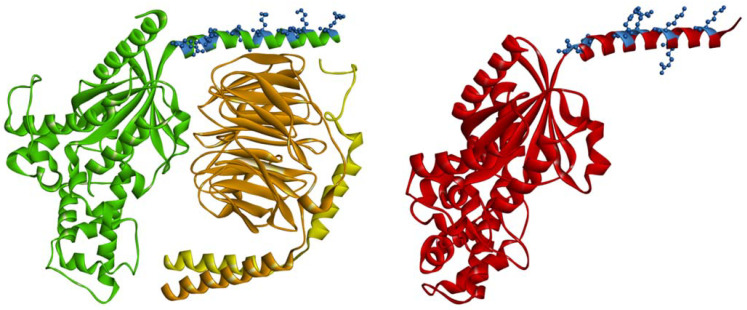
Localization of positively charged amino acid residues (blue balls and sticks) of the N-terminal fragment in the context of the tertiary and quaternary structure of Gαsβ_1_γ_2_ heterotrimer and Gαi_1_ subunit (red). The Gαs is colored green; the Gβ_1_ is orange; the Gγ_2_–yellow. The diagram was generated using coordinates from the PDB: 6X18 and 6CRK and visualized with BIOVIA Discovery Studio 4.0.

The number of basic amino acids in Gα subunits, depending on the Gα family, varies between 6 and 10 (Figure 1). They are localized within amino acids 8–55 from the N-terminus in the helical region of the protein. Electrostatic interactions with the plasma membrane are possible even when Gα is bound to Gβγ, because the polybasic motifs are localized on that part of the helix that is not directly involved with Gβγ interaction (Figure 2) [37,38,97]. It is postulated that N-terminal polybasic clusters are more pronounced in membrane localization of the non-myristoylated Gα families (Gαs, Gαq/11, and Gα12/13) where they not only compensate for myristoylation but also act together with Gβγ and palmitoylation in targeting the heterotrimer to the plasma membrane [37,38,90]. Most of these proteins contain two polybasic subclusters (basic patches). Cell-based studies have shown that mutations of lysine and arginine in the polybasic region of Gαs and Gαq strongly affect membrane localization of these proteins [90]. For Gαs, replacement of four lysine residues (at positions: 24, 25, 28, and 32) with glutamine residues was sufficient to cause a significant decrease in membrane binding, whereas for Gαq, nine basic residues had to be mutated to achieve a similar effect. The polybasic cluster also mediates membrane localization of the complete heterotrimer of G protein [90]. Furthermore, the lysine and arginine mutants of Gαs and Gαq showed a defect in membrane localization even when Gβγ dimer was co-expressed. However, it appears that only the first basic patch in the N-terminus (the three basic amino acids downstream the anchors) of Gα plays a key role in membrane attachment. As shown for Gαq, the replacement of these three basic residues with glutamic acid residues, to switch the charge of the side chains, strongly disrupted membrane localization [90]. The polybasic mutants that were defective in membrane binding were also defective in signaling. Other studies have shown that N-terminal basic amino acids of Gαq not only contribute to membrane attachment but also participate in its segregation within membrane domains [37]. Mutant containing glutamine or alanine substitutions at arginine residues 27, 30, 31, and 34 (the second basic patch) was identified to fail in mediating signaling, even though Gαq was localized at the plasma membrane. However, the nanoscale plasma membrane distribution of this mutant differed from that of wild-type Gαq. Thus, the N-terminal basic residues of Gαq could affect signaling function independently of their general role in strengthening plasma membrane attachment. Mutational analysis of other Gαq/11 family members, namely Gα14 and Gα16, revealed that replacement of the N-terminal polybasic cluster had minimal effect on the plasma membrane localization of Gα14 but not of Gα16 [91]. When Gα16 was deprived of most of the positive charge in the N-terminus, its amount was markedly reduced in the membrane fraction, and it exhibited minimal functional activity. The polybasic region in Gα14 includes several additional positively charged amino acids (nine in total) compared to the polybasic cluster of Gα16 (six in total). Hence, the role of the polybasic motifs can differ even among members of the same Gα family, and it seems that not only their number but also their location within N-terminus is crucial.

The polybasic clusters found in myristoylated Gα families are smaller than those found in non-myristoylated Gα families and are usually reduced to 6–7 residues (Figure 1) [38]. An exception is Gαz, which is an atypical member of the Gαi family and has a more extensive positive patch but undergoes phosphorylation on two residues within the polybasic motif [98]. All Gα subunits have negative charges introduced in the N-terminal helix close to the N-terminus region. Positively charged patches can be at least partially compensated by a negative charge in myristoylated Gα [38]. This hypothesis was further confirmed by calculating the electrostatic potential of the Gαt as the entire surface of this subunit is predominantly negative [99]. Consequently, the membrane affinity of transducin to negatively charged lipids is relatively weak. After association with Gβγ, the distribution of negative charges on Gαt surfaces changes. Thus, the membrane interactions of the dissociated transducin differ from those of the heterotrimer. However, under physiological conditions, the electrostatic contribution of the Gαt heterotrimer to the membrane affinity is rather insignificant [99]. The surface of the heterotrimer is mostly negatively charged, except for the small area around the lipid anchors attachment points of Gγ and Gαt. Kosloff and colleagues hypothesized that the polybasic motifs and myristoylation have complementary roles as a targeting signal [38]. It is suggested that for myristoylated Gα families, membrane attachment is likely to be controlled exclusively by the lipid anchors rather than the positive charge in the N-terminus region.

Interestingly, a recent report addressing membrane localization and distribution of the Gαi family members, namely Gαi_1_, Gαi_2_, and Gαi_3,_ showed that the N-terminal polybasic cluster affects its interaction with the plasma membrane [30]. Nevertheless, the electrostatic potential maps of Gαi subunits are similar and contain negative patches that produce the Gαt membrane repulsion [38]. The cell-based fluorescence recovery after photobleaching (FRAP) experiments revealed that the lateral diffusion and nanoscale membrane distribution of these subunits differ, despite high sequence similarity. The lateral mobility of Gαi_1_ and Gαi_3_ was found to be similar; however, the diffusion coefficient of Gαi_2_ was much higher. Although the Gαi subunits showed high similarity in the N-terminal polybasic regions, two seemingly insignificant substitutions are observed (substitution of arginine for lysine or vice versa in positions 21 and 32). Lysine and arginine differ in their geometric structure and possible interactions. Arginine can form a higher number of electrostatic interactions such as salt bridges and hydrogen bonds; hence, it presumably results in stronger interactions than those generated by lysine [100,101]. However, the nature of the interaction of arginine with the membrane can lead to a local distortion of the bilayer around this residue [102]. This distortion is manifested as a high level of local water penetration inside the membrane and can lead to a decrease in the thickness of the bilayer as well as affect long-range interactions [102]. The reduced membrane mobility of Gαi_1_ corresponds to the presence of a larger number of arginine residues in the polybasic motif compared to that in the other two Gαi subunits [30].

General takeaways from studies of the role of polybasic motifs in Gα subunits on its membrane interaction are that (i) the sequence of the N-terminal polybasic region is important for plasma membrane attachment and distribution; (ii) the role of the polybasic motifs can differ even among members of the same Gα family; (iii) the polybasic motif in myristoylated Gα families influences membrane interaction, not only anchors; (iv) the number and distribution of lysine and arginine residues are important; (v) association with Gβγ affects the interaction with the membrane; and (vi) a combination of multiple covalent lipid modifications occurs. The unique electrostatic properties derived from the distribution, type of residue, or its environment provide different membrane-binding affinities and contribute to the specificity of their protein–lipid interactions.

## 3. Looking from the Plasma Membrane Side; What Makes Gα Proteins Associate with It?

One of the potential advantages of having multiple membrane-targeting motifs for membrane anchoring is the capacity for easy regulation of membrane associations. For Gα proteins, lipidation is responsible for a long-lasting interaction with the plasma membrane, whereas polybasic-mediated electrostatic complementarity can be dynamic and subject to regulation through changes in the membrane lipid composition and changes in the electrostatics of the polybasic region. Studies on the role of the membrane in modulating the function of membrane-bound small GTPases revealed that specific protein surfaces containing positively charged residues interact directly and transiently with the membrane [103]. Consequently, the proteins adopt distinct and specific orientation with respect to the membrane. These interactions can be modulated by the conformational state of the protein, and conversely, the membrane modulates the function of proteins by affecting their conformation. In contrast to the well-understood phenomenon of the nucleotide-dependent conformational change of G proteins and its role in biological activity, the functional significance of conformational changes in Gα originating from interactions with the membrane is poorly understood and lacks data.

Nuclear magnetic resonance (NMR) studies showed that lysine and arginine, belonging to the polybasic regions of membrane-associated proteins, are embedded in the membrane and reside at the interface between membrane hydrophilic and hydrophobic regions [104]. Thus, positively charged amino acids interact directly with the negatively charged headgroups of acidic phospholipids [96,105]. Compared to the outer leaflet, the inner leaflet of the mammalian plasma membrane is not only enriched in phosphatidylethanolamine (PE) but also in anionic headgroups phospholipids, including phosphatidylserine (PS), phosphatidylglycerol (PG), phosphatidic acid (PA), and phosphatidylinositol (PI) [106,107,108]. In addition, a small proportion of the membrane PI is phosphorylated at the 3-, 4-, and/or 5- positions to generate inositides: PtdIns(4)P, PtdIns(3,4)P_2_, PtdIns(4,5)P_2_, and PtdIns(3,4,5)P_3_. PE is favorably distributed in the inner leaflet of the plasma membrane, because its inverted conical molecular shape can induce a negative curvature of the leaflet [109]. Additionally, the membrane areas of the negative curvature are locally enriched in PtdInsP_2_ lipids [110,111].

Multivalent membrane anionic lipids generate a surface potential that attracts and binds positively charged ions. Interestingly, PA also plays an essential role in the binding of proteins containing polybasic clusters and has also been proposed to act as an important player in the transmission, amplification, and regulation of a large number of intracellular signaling events and cellular functions (review: [112,113,114]). The unique feature of PA, compared to other phosphoglycerides, is its phosphomonoester link to a small, negatively charged, phosphate headgroup. At the molecular level, PA interaction with arginine and lysine residues would be solely electrostatic in nature, but hydrogen bonds can also be formed between PA and both basic amino acids in a lipid bilayer [115]. Lysine, however, seems to be more effective in docking to PA headgroups than arginine. This is most probably due to the substantial delocalization of charge in the arginine guanidinium group and because it is a weaker hydrogen bond donor [115].

Selectivity of polybasic clusters present in peripheral membrane proteins toward the headgroup of acidic phospholipids is difficult to predict because it depends on the three-dimensional protein structure surrounding the charged residues. Proteins, however, might differentiate between PS, PA and the family of phosphoinositides, and the affinity for particular phospholipids is diverse [93,116,117,118]. For a number of proteins, e.g., Ras superfamily GTPases, the most targeted phospholipids are PtdIns(4)P and PtdIns(3,4)P_2_, even though they are the fewest in the membranes [93,119,120]. Selectivity for PIs over PS or PA is attributable to its higher negative charge, resulting from the presence of polyanionic lipid headgroups. However, this cannot be taken as a general rule [121]. Polybasic proteins may have a preference for other anionic lipids beyond simple electrostatically driven interactions. For instance, K-Ras4B, which contains six lysine residues adjacent to a farnesylated cysteine residue, has been shown to preferentially interact with PS [122].

Regarding the interactions of negatively charged phospholipids with Gα proteins, several studies have been conducted on PS, which is the most abundant. Unfortunately, little is known about the function of PA, PG, or PIs in the localization of G proteins in the plasma membrane. Most available molecular-level data on G protein interactions with lipids are derived from studies on transducin, but this protein resides in a highly specialized membrane environment (rod outer segments, [ROS]) (review: [123]). By contrast, GPCRs and G proteins outside of the visual system function in different membrane environments at much lower local concentrations, and finally, each G protein heterotrimer is a slightly different one. The cytoplasmic surface of the ROS is composed mainly of PE and PS and includes large amounts of cholesterol [124]. It has been shown that negatively charged PS is more critical for heterotrimeric Gαt membrane anchoring than for the monomeric Gαt [125]. Electrostatic interactions, especially of Gβγ, further enhance the membrane binding to a negatively charged surface containing a small cluster of acidic PS lipid [126,127]. Furthermore, PS also plays an important role in forming the complex between activated rhodopsin and Gαt heterotrimer, most likely by influencing the rhodopsin structure and simultaneously providing a platform for G protein anchoring to the membrane [125,128]. After the activation of rhodopsin and G protein, phospholipids are redistributed within the disc membrane, and monomeric Gαt undergoes translocation from the center to the disc periphery, which differs in lipid composition [129,130,131]. The conclusion from these studies is that heterogenic lipid domains exist in the ROS disc lipid bilayer and that Gαt subunits are distributed within these domains in a manner dependent on their functional state.

PS also appears to be important for the functioning of the Gαi protein family. Interactions of monomeric Gαi_1_ with model membranes containing phosphatidylcholine (PC) and PS lipids strengthen with increasing proportions of PS [132]. Notably, the binding of Gαi_1_ to acidic lipids is modulated by its lipid anchors. It was observed that the myristoylated and palmitoylated Gαi_1_ has a lower affinity to the PS membrane than unlipidated subunit or protein with only myristic acid attached. In addition, higher proportions of PE reduced the binding of monomeric Gαi_1_ to the lipid bilayer, whereas the affinity of its heterotrimeric complex increased [133]. These studies suggest that lipid preferences of Gαi_1_ are contrary to the preferences of the full G protein complex [133,134]. The data currently available establish that most, if not all, heterotrimeric G protein complexes show membrane localization different from that of monomeric Gα subunits [31,134,135].

Negatively charged lipids have been found to modulate G protein interactions with GPCRs and signal transduction properties. However, almost all our knowledge on the role of lipids in G protein binding to GPCRs is derived from rhodopsin and adrenergic and neurotensin receptors, and only a few lipids have been investigated. Although several structures have been solved for GPCR–G protein complexes, structural studies of the complex in a physiological lipid membrane environment are still lacking, and the role of lipids in this binding is not fully understood (most of the solved structures were determined in detergent micelles and/or in the presence of stabilizing antibodies/nanobodies and engineered Gα subunits) [136,137,138,139,140]. In particular, fluorescence studies revealed that local membrane charge differentially modulates the interaction of β_2_ adrenergic receptor (β_2_AR) with competing G proteins [141]. Negatively charged PG or PS membrane (nanodiscs) impairs coupling of β_2_AR to Gαi_3_β_1_γ_2_ and facilitates coupling to Gαsβ_1_γ_2_. Another study using native mass spectrometry for the identification of endogenous lipids bound to selected GPCRs demonstrated that PtdIns(4,5)P_2_ stabilizes the complex between the activated receptor and certain G proteins [142]. According to this study, the β_1_-adrenergic receptor (β_1_AR), the neurotensin (NTSR1) receptor, and the adenosine A_2A_ receptor (A_2A_R) exhibited the capacity for binding with high affinity to PtdIns(4,5)P_2_, thus implying the presence of preferential binding sites for this lipid. The association of lipids was found to be higher when β_1_AR was in a complex with mini-Gαs (engineered, lacking the α-helical domain) than for the receptor alone. PtdIns(4,5)P_2_, but not PS, enhances mini-Gαs coupling with the receptor. Similarly, the interaction of mini-Gαs with the A_2A_ receptor is stabilized significantly in the presence of PtdIns(4,5)P_2_ when compared with PS. By contrast, PtdIns(4,5)P_2_ has no effect on the coupling of mini-Gαi and mini-Gα_12_ to β_1_AR; however, it improved the coupling of Gαiβγ to the active NTSR1 receptor and GTPase activity of Gαi [142]. Recent cryo-EM structures of lipid bilayer-bound complexes of NTSR1 and Gαi_1_β_1_γ_1_ proteins showed clear interactions of Gαi_1_ with the lipid bilayer [143]. In these structures, the N-terminal helix of Gαi_1_ is located close to the lipid bilayer formed by PC and PG lipids (circularized nanodiscs), and high lipid density is observed at the myristoylation site and around the positively charged sidechains of arginine and lysine. This observation agrees with an earlier finding that negatively charged PG strengthens the interaction between NTSR1 and the Gαqβ_1_γ_1_ heterotrimer and markedly increases nucleotide exchange at Gαq [144].

Lateral heterogeneity of the membranes promotes the formation of molecular clusters or domains with sizes ranging from nanometers to micrometers due to nonideal mixing of the different membrane components (lipid rafts and micro/nanodomains) [145,146]. Lipid rafts are heterogeneous, highly dynamic, sterol- and sphingolipid-enriched domains that compartmentalize cellular processes [147,148]. However, not only sphingolipids and cholesterol but also ceramides, glycosphingolipids, and phosphoinositides contribute to lipid segregation into domains [111]. The charged proteins interacting at the interfacial region with the charged lipids have been shown to generate clusters and can induce changes in the nanoscale organization of the lipid membranes and the formation of nanodomains [111,149,150]. Nevertheless, the underlying molecular mechanism is not fully understood. Unquestionably, membrane domains have different chemical compositions and/or physical properties as compared to their surrounding lipid environment, and the asymmetric distribution of lipids between the two leaflets of the plasma membrane bilayer is a prevalent and fundamental feature of living cells [151,152]. Recent data revealed that the inner leaflet contains largely low-melting unsaturated lipids that are not amenable to ordered domain formation, although the high abundance of long-chain sphingolipids species in the outer leaflet may promote domain coupling between the leaflets [153]. The ultimate organization of the membrane is then a combination of these membrane-intrinsic effects and extrinsic inputs such as protein scaffolds and cytoskeletal dynamics [154,155]. The data currently available establish that local lipid composition, membrane thickness, and curvature stress in the membrane, together with the particular physical properties of the proteins, are important factors that affect its membrane partitioning.

Early conducted experiments showed the affinity of some Gα subunits to membrane regions with specific features; as shown, various G proteins accumulate in detergent-resistant membrane fractions [156]. G protein monomers or heterotrimers were docked to mimicking raft domain liposomes. Experiments showed that the myristoylation and palmitoylation of Gαi subunits are necessary and sufficient for protein attachment with such membrane areas [156]. Preferential localization in lipid rafts was confirmed for Gαi and Gαs subunits, although a small population of these subunits is also present in caveolae [157]. The specific interaction of Gαq with caveolin allowed researchers to conclude that caveolin cavities are the major region of residence of this subunit [157]. In our research, we focused on the localization and protein interactions of the dopamine signal transduction pathway: D_1_ and D_2_ receptors and cognate G proteins, Gαs and Gαi_1-3_ subunits [30,31,135,158]. To study the mutual distribution of the receptor-G protein complex, we used microscopy techniques to measure the distance between the proteins and their lateral diffusion. The lateral diffusion of G proteins indicates their distinct localization. The diffusion of the Gαi_3_ subunit is much faster than that of the Gαs subunit [135,158]. Our study showed that Gαs localizes to the membrane regions with significantly less fluidity as compared to Gαi_3_. Gαi_3_ prefers more mobile membrane fractions. Interestingly, co-expression of the studied proteins in a system with the dopamine D_1_ receptor resulted in significant changes in the diffusion of both subunits, but these changes had a distinct outcome. In the next step of the study, we verified the effect of reduced concentrations of important lipid membrane components such as cholesterol and sphingolipids [135]. To deplete the concentration of cholesterol or sphingolipids in the cell membrane, we treated HEK293 cells with β-cyclodextrin or fumonisin B_1_, respectively. The extraction of cholesterol leads to disruption of ordered domains such as lipid rafts and caveolae [159,160]. The results indicate the different sensitivities of the Gαs and Gαi_3_ subunits to cholesterol and sphingolipid concentrations. The Gαs subunit prefers solid-like domains that are insensitive to cholesterol and the structure or composition of lipid rafts. In contrast, the Gαi_3_ subunit favors localizing to more fluid regions of the membrane together with lipid rafts. Furthermore, Gαs subunit membrane distribution is affected by both the D_1_ receptor and the Gβγ dimer. The presence of the Gβγ dimer directs the associated Gαs subunit away from the liquid-ordered domain regions.

Previous reports show a direct role of the Gγ subunit in heterotrimer activation [161,162,163]. The significance of the Gγ subunit for the localization and assembly of the heterotrimeric G protein complex was also demonstrated [17,18,164]. We also tested the effect of two distinctively prenylated Gγ subunits, namely Gγ_2_ and Gγ_9_, on heterotrimer localization. We observed that the geranylgeranylation of Gγ_2_, together with close positively charged residues, provides a higher affinity for binding to the plasma membrane as compared to the farnesylated Gγ_9_ subunit. In our experiments, the formation of a complex of the Gα subunit with the Gβγ dimer resulted in accelerated lateral diffusion in all tested heterotrimers. However, the increase in diffusion coefficient was a characteristic of the complex composition [31]. In contrast to previous reports, we demonstrated that both the Gβγ dimer and the Gα subunit determine the final membrane localization of the full heterotrimer. Individual heterotrimers present diverse localization patterns because of the interaction of lipid anchors and charged regions of protein components with the cell membrane.

Despite the vast amount of research on the coupling specificity of receptor-G protein, little is known about the selectivity of receptors to similar subunits of a single subfamily. As the individual components of the heterotrimer are important for its localization, we decided to test the effect of the membrane organization of alike heterotrimers on dopamine D_2_ receptor signaling [30]. We focused on the functional selectivity of the D_2_ receptor toward Gαi_1-3_ subunits. All these subunits are N-myristoylated and S-palmitoylated and share high sequence identity. Despite the similarities, we observed significant differences in lateral diffusion of each subunit, which affected signaling after receptor stimulation. Thus, protein–lipid interactions appear to be crucial in GPCR-dependent cell signaling. Therefore, the lipid environment should be considered as one of the determinants for G protein coupling selectivity.

## 4. Does It Matter to Us? Why Is It Worth Researching?

In the last decades, scientists have concentrated on understanding how GPCRs work, which is perfectly understandable, as their role in the proper functioning of every mammalian organism is crucial. The primary effectors of GPCRs are the heterotrimeric G proteins, and they can also be substantially affected by the lipid composition of the plasma membrane. The functions of cell membranes far exceed enclosing and compartmentalizing cells and organelles. There is growing knowledge that cell membranes regulate transport, provide sites for enzyme binding and catalysis, anchor cytoskeleton elements, and influence signal transmission from GPCR to G proteins [165,166]. Not only GPCR activation but also cell membrane composition has a significant effect on signaling mediated by G proteins, as it creates room for molecular events to occur. Diet-, nutrient-, or drug-treatment-induced changes in lipid composition can alter biophysical properties of plasma microdomains and, as repercussion changes, in the occurrence and actions of coexisting proteins. G protein and plasma membrane lipids are essential for cell physiology. Disturbance of their actions leads to metabolic, cardiovascular, neurodegenerative, and oncological diseases. The involvement of G protein–lipid interactions has been proven in some of these abnormalities.

The role of membrane lipids and their importance in signaling through G proteins has been well documented in the example of interaction between rhodopsin and Gαt protein [123,167]. However, the environment in which the visual-signal transmission components are found is unique and cannot be directly translated into signal transmission through nonvisual receptors. An interesting study shows the influence of lipid environment on G protein signal transmission related to the neurotensin receptor—one of the receptors with high pharmacological potential. It is believed that this can be a target in the treatment of neurodegenerative (schizophrenia, Parkinson’s disease), metabolic (obesity), and cancer diseases. Inagaki et al. showed that in reconstituted nanodiscs, increasing the negative charge density (three different lipid environments) induced a change in G protein coupling to the receptor. The authors claim that in negatively charged lipids, the GDP/GTP nucleotide exchange at the Gαq protein is favored, and the authors exclude the influence of the studied lipid composition on the conformation of the neurotensin receptor binding site. This is direct evidence for the influence of lipid composition on G proteins; however, the experimental environment was artificial. The lipid composition differs from tissue to tissue, and even within one cell, the composition of the cell vicinity is heterogeneous [144].

The central nervous system is the second-largest location of lipids in the body after adipose tissue. It is therefore no wonder that lipids are important in the formation and treatment of central nervous system disorders. Gαs protein localization in the plasma membrane plays a significant role in the development of depressive disorders, and the success of antidepressant therapies is linked to the actions on G proteins. There is, however, no conclusive information on the changes in the amount of individual Gα subunits in adult suicide victims and in studies on model organisms, and neither on the effect of drug treatment nor on the amount of these proteins. Nevertheless, long-term antidepressant treatment and electroconvulsive therapy appear to increase the cAMP concentration in certain regions of the rat brain in a Gαs-dependent manner [168]. Lipid rafts in which G proteins accumulate influence their signaling potency in different ways, depending on the Gα protein type. Analysis of multiple studies showed that in depressed individuals, Gαs proteins were localized in lipid rafts, where they were less likely to couple to adenylyl cyclase. Moreover, antidepressant drugs have been shown to affect membrane microenvironments in a way such that the amount of lipid raft-associated Gαs subunits was reduced, which resulted in increased cAMP accumulation in suicide cases [168,169]. This was confirmed in cell model experiments. Antidepressant treatment reduced the amount of Gαs in lipid rafts without affecting the level of expression of this protein [170].

Another example of neurological abnormality resulting from disturbances in the plasma membrane and associated with GPCRs signaling is Alzheimer’s disease (AD). It is a well-documented example of linking membrane lipids to the metabolism of proteins related to AD, especially amyloid β-peptide generation and aggregation [171,172,173]. A growing body of research indicates that cholesterol, a major regulator of lipid organization in the membrane, and sphingolipids are involved in the process of AD pathogenesis [174,175]. On the other hand, many reports present evidence that different GPCRs are related to the pathological progression of AD [176,177,178,179]. To date, there is no direct evidence that the interaction of G proteins with the membrane is involved in the development of this disease, but the unquestionable influence of cholesterol on its biogenesis and the influence of cholesterol on the signal transmission through G proteins [135] allows us to hypothesize that the interaction of G proteins with lipids is important in the development of AD. Cholesterol also appears to play a leading role in the progression of abnormalities of the cardiovascular system. Information linking it with G proteins emerges from studies on elderly hypertensives. In the erythrocytes of this population group, the level of membrane phospholipids was decreased as compared to that in healthy subjects, and the level of cholesterol was increased. Complementary studies showed that the amount of membrane-associated G proteins was reduced [180]. The authors of this experiment claim that changes in membrane lipid composition affect the localization and actions of G proteins. Another set of experiments performed on red blood cells showed that the ratio of saturated-to-unsaturated fatty acids in patients with type 2 diabetes is higher than that in healthy subjects, and again, G protein levels show variation between the two groups [181].

Cancer, which was the second leading cause of death globally in 2020 according to the WHO, is another example of the influence of the lipid membrane on the condition of the body [182]. Lipid membranes of cancer cells and healthy cells differ significantly [183]. Unlike healthy cells, cancer cells show exposure to PE and PS phospholipids on the exoplasmic part of the membrane. Another example of change observed in cancer cells is increased cholesterol content, which causes additional stiffening of the cell membrane [184]. It has been shown that medicines used for the treatment of solid tumors and leukemias reduce the formation of the nonlamellar phase and thereby reduce the levels of membrane-associated G proteins and PKC, as observed in heart and brain plasma membranes, consequently disrupting oncogenic signaling [185].

In the classical sense, the role of G proteins is restricted to a response to membrane receptor activation. This function is very well described and has been shown to regulate many intracellular processes. Post-translational modifications of membrane proteins and lipid membrane composition influence the intracellular localization of G proteins. Changes in the intracellular localization of G proteins and the role of lipids in these processes have not been sufficiently understood. Increasing evidence suggests that the involvement of lipids in the regulation of signal transduction provides a broader and more dynamic picture, in which many more factors than once thought are co-responsible for the final effect on GPCRs. As the above considerations suggest, this effect is largely dependent on the spatiotemporal localization of lipids.

## Figures and Tables

**Table 1:** Gα subunit expression and well-defined effector partners.

Family	Member	Distribution	Major Signaling Role
Gαs	Gαs_(S)_ Gαs_(L)_	ubiquitous	adenylyl cyclase (+)
	Gαs_(XL)_	neuroendocrine cells	
	Gαolf	olfactory sensory neurons, striatum	
Gαi/o	Gαi_1_, Gαi_2_, Gαi_3_	ubiquitous	adenylyl cyclase (−)
	Gαo_1_, Gαo_2_	neurons, heart, neuroendocrine cells	
	Gαz	rod and cone cells of the eye	
	Gαt_1_, Gαt_2_	taste cells	phosphodiesterase (+)
	Gαg	neurons and platelets, adrenal chromaffin cells, neurosecretory cells	
Gαq/11	Gαq	ubiquitous	phospholipase C (+)
	Gα11	ubiquitous	
	Gα14	kidney, lung, liver, spleen	
	Gα15	hematopoietic cells	
	Gα16	hematopoietic cells	
Gα12/13	Gα12	ubiquitous	Rho GTPases (+)
	Gα13	ubiquitous	

## Data Availability

Not applicable.
